# Hepatic methanethiol oxidase (MTO) activity of mice requires adequate dietary supply of copper

**DOI:** 10.1016/j.redox.2026.104384

**Published:** 2026-09-06

**Authors:** Niklas Krafczyk, Kristina Lossow, Lara Biegel, Nora Meinhardt, Maria Schwarz, Hans Zischka, Holger Steinbrenner, Anna Patricia Kipp, Lars-Oliver Klotz

**Affiliations:** a Institute of Nutritional Sciences, Nutrigenomics Section, Friedrich Schiller University Jena, Jena, Germany; b Institute of Nutritional Sciences, Department of Nutritional Physiology, Friedrich Schiller University Jena, Jena, Germany; c Institute of Toxicology and Environmental Hygiene, Technical University Munich, School of Medicine and Health, Munich, Germany; d Institute of Molecular Toxicology and Pharmacology, Helmholtz Center Munich, German Research Center for Environmental Health, Neuherberg, Germany

**Keywords:** Copper, Selenium, MTO, H_2_S, SELENBP1, Wilson's disease, Sexual dimorphism, HepG2

## Abstract

Selenium-binding protein 1 (SELENBP1) catalyzes the oxidative conversion of methanethiol to hydrogen sulfide, hydrogen peroxide and formaldehyde. Methanethiol oxidase (MTO) activity of recombinant human SELENBP1 was shown to require copper (Cu) ions, whereas selenium (Se) was largely dispensable. However, the impact of Cu and Se on MTO activity in mammals has not been explored so far. To investigate whether SELENBP1 levels and MTO activity in mice are affected by dietary availability of trace elements, liver samples of C57BL/6JRj mice held on diets deficient in Cu and/or Se for eight weeks were analyzed, as compared to mice fed the control diet with adequate trace element supply. Dietary Cu deficiency always resulted in diminished hepatic MTO activity, as detected by methanethiol-derived production of hydrogen sulfide. The effect of Cu deficiency was more pronounced in male than in female mice. Se deficiency only slightly, and solely in males, lowered MTO activity. Another sex-specific effect was observed with hepatic SELENBP1 levels: whereas diets deficient in Cu and/or Se increased SELENBP1 levels in males, they were decreased in female mice under Cu-deficient conditions. In addition to dietary Cu deficiency, MTO activity and SELENBP1 levels were explored in a rat model of Wilson's disease, which is characterized by hepatic Cu accumulation. Hepatic MTO activity was not altered in male and female rats under conditions of excess intrahepatic Cu, as long as the liver was not severely damaged. Notwithstanding the relatively low number of 5-6 animals included in each group, our data allow for the conclusion that hepatic MTO activity depends on adequate dietary Cu supply, whereas excess Cu does not further enhance the MTO activity of SELENBP1.

## Highlights

• Murine selenium-binding protein 1 is a copper-binding methanethiol oxidase (MTO).
• Dietary Cu deficiency leads to lower hepatic MTO activity in mice.
• Dietary selenium has only a minor impact on hepatic MTO activity.
• Hepatic Cu excess in a Wilson's disease rat model does not increase MTO activity.
• Adequate dietary Cu supply is necessary and sufficient for hepatic MTO activity.

## Graphical abstract

**Figure:**
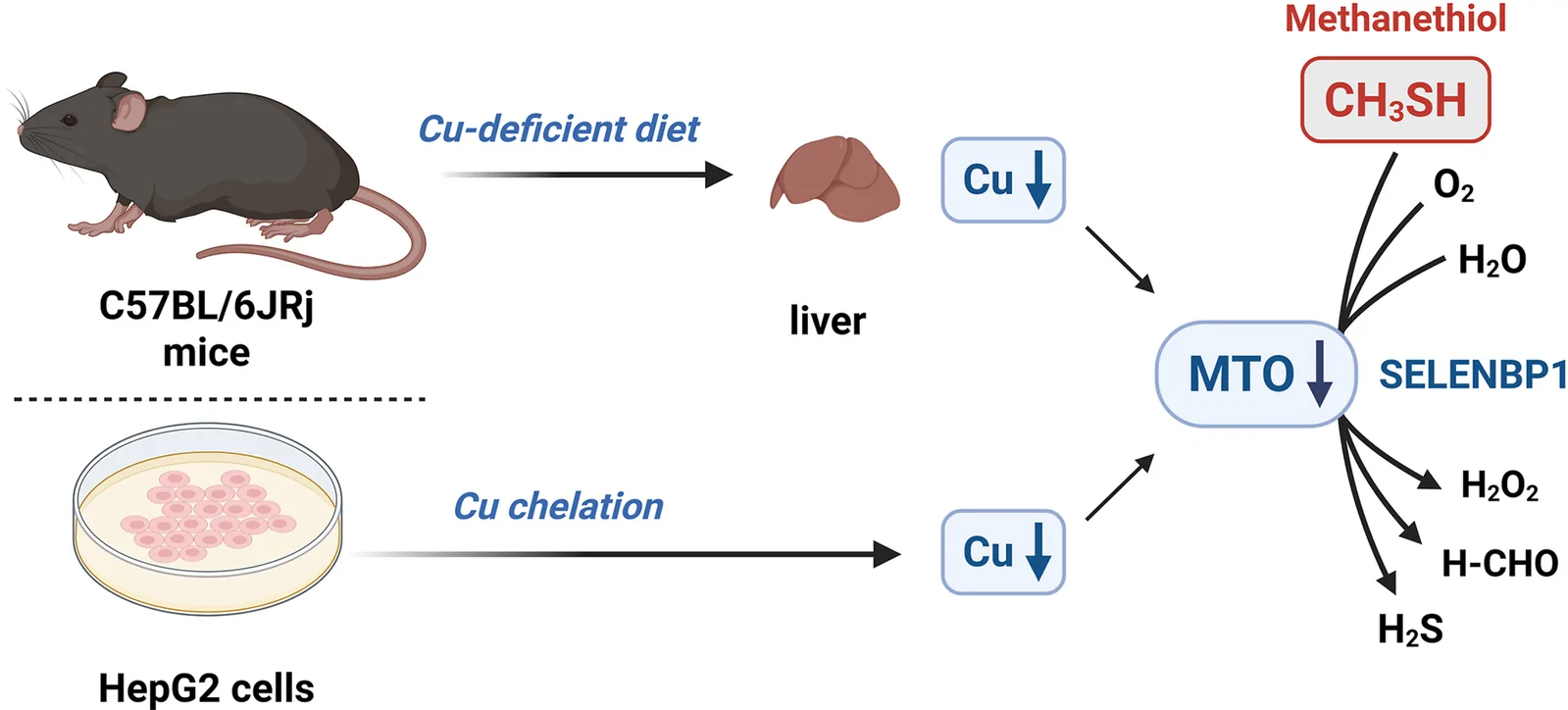

## 1 Introduction

Selenium (Se) is an essential trace element (TE) and, as selenocysteine, an integral constituent of 25 selenoproteins in humans, including glutathione peroxidases and other selenoenzymes [1,2]. Selenium-binding protein 1 (SELENBP1), in contrast to selenoproteins, was identified due to its ability to bind Se species at supraphysiological concentrations, through interaction with cysteine residues [3,4]. In humans, SELENBP1 is ubiquitously expressed, with highest levels in the intestine, the liver and the lungs. It has been proposed to act as tumor suppressor, based on its inhibitory and supporting effects on cell proliferation and differentiation, respectively, and its pronounced downregulation in various malignancies [5,6].

In 2018, SELENBP1 was demonstrated to be a methanethiol oxidase (MTO), catalyzing the oxidative conversion of methanethiol (MT) to hydrogen sulfide (H_2_S), hydrogen peroxide (H_2_O_2_) and formaldehyde [7]. Presumably, this enzymatic activity represents its major physiological assignment in mammals, as MT is a volatile toxic intermediate in the metabolism of sulfur compounds that can be produced by intestinal microbiota as well as by endogenous cellular processes [6,8,9]. SELENBP1 may thus protect the organism from adverse effects of high MT levels that are mainly attributed to inhibition of electron transfer in the mitochondrial respiratory chain [10]. In accordance with the downregulation of SELENBP1 in tumors, elevated MT levels were detected in breath and flatus of lung and colon cancer patients, respectively [11]. Moreover, increased serum MT levels were observed in patients with liver pathologies [[12], [13], [14]].

Despite its Se-binding capacity, Se was not required for MTO activity of recombinant human SELENBP1 [4]; rather, we recently demonstrated that the presence of copper (Cu) ions is essential for its MTO activity, which was true also for the recombinant *Caenorhabditis elegans* (*C. elegans*) ortholog, SEMO-1 (SELENBP1 ortholog with MTO activity [15]), whereas other TEs were dispensable [4]. These findings were in line with earlier metalloproteomic studies that listed SELENBP1 among hepatic proteins potentially binding Cu [16,17].

As an essential TE to mammals, Cu is required for a multitude of cellular processes, including mitochondrial ATP production, antioxidant protection, iron homeostasis and xenobiotic metabolism. Owing to the redox properties of Cu ions, shifting between cupric (Cu^2+^) and the reduced cuprous (Cu^+^) state, most of the almost 30 known Cu-dependent enzymes in humans are oxidoreductases, including the MTO SELENBP1 [18]. Even though dietary Cu deficiency or excess rarely occur in humans, two genetically determined disorders underscore the critical need for maintaining Cu homeostasis: Wilson's disease (WD) is caused by loss-of-function mutations in the *ATP7B* gene, encoding the hepatic Cu-transporting ATPase, ATP7B. The resulting Cu overload in the liver and subsequently in the brain causes liver cirrhosis and neuropsychiatric pathologies. By contrast, patients suffering from Menkes disease exhibit diminished intestinal Cu resorption due to mutations in the *ATP7A* gene, resulting in neurodegeneration and atypical connective tissue formation [[18], [19], [20], [21]].

**Fig. 1:**
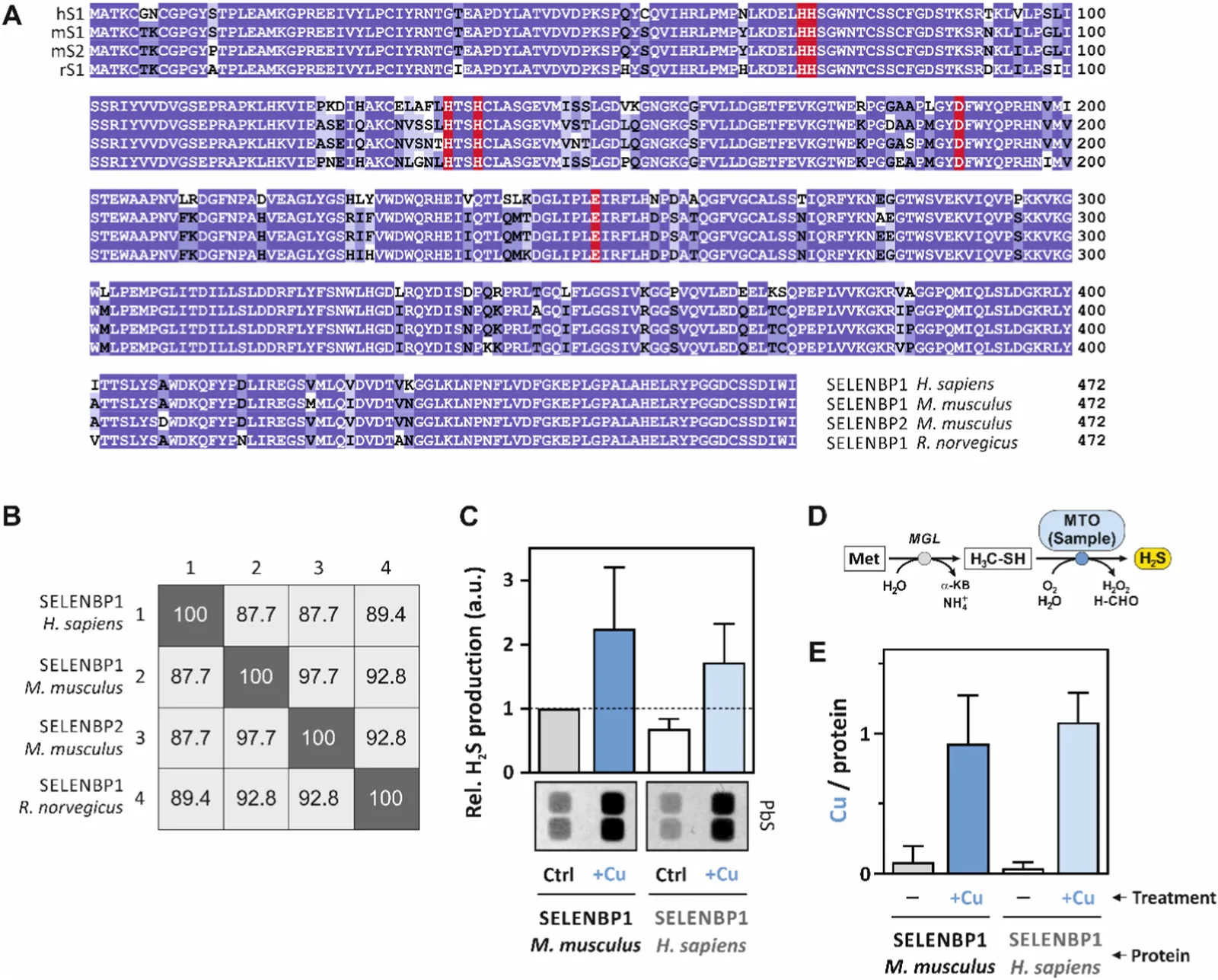
Comparison of human and rodent selenium-binding proteins. (A), Alignment of *H. sapiens* SELENBP1 (hS1; UniProt entry Q13228), M. musculus SELENBP1 (mS1; P17563) and SELENBP2 (mS2; Q63836), as well as R. norvegicus SELENBP1 (rS1; Q8VIF7). Residues identical between sequences are indicated in blue; those shown in white font represent positions that are identical across all four sequences. Residues previously identified as required for methanethiol oxidase activity of human SELENBP1 [4] are shaded in red. **(B),** Percent identity matrix analysis between human, murine and rat SELENBPs, generated with Clustal 2.1. **(C),** Methanethiol oxidase (MTO) activity of recombinant Strep-tagged murine and human SELENBP1, without or with additional Cu(II) added during affinity purification. Three independent experiments were performed in duplicate each. Bars show means + SD of densitometric analyses of lead sulfide (PbS) traces, with values for murine SELENBP1 set to 1. PbS precipitates for one representative experiment are shown under the bar graph. **(D),** Schematic representation of MTO assay principle: Methionine γ-lyase (MGL) catalyzes the formation of methanethiol (H_3_C-SH) along with α-ketobutyrate (α-KB) and ammonium through degradation of methionine (Met). Methanethiol is then oxidized by a methanethiol oxidase (MTO) to generate hydrogen peroxide, formaldehyde and hydrogen sulfide (yellow), which is detected in the assay as a PbS precipitate. **(E)**, Cu content of murine and human SELENBP1 following treatment with CuCl_2_ as in (C). Bars show means of three independent experiments + SD.

In *C. elegans* held under conditions of Cu abundance or Cu deficiency, *ex vivo* analysis of MTO activity supported the notion of a direct link between Cu supply and MTO activity [4]. However, it is currently unknown to what extent the endogenous MTO activity in mammals is affected by Cu availability. The present study addresses this question using rodent models of dietary Cu deficiency and genetically determined hepatic Cu excess. The homologous SELENBP/MTO proteins in humans, mice and rats show a high degree of sequence identity, with conservation of major features, including two metal-binding motifs and the amino acid residues D189 and E252 that we have previously identified as being involved in Cu binding [4] (Fig. 1A and B). We here demonstrate that hepatic MTO activity increases with dietary Cu content in mice, whereas no further elevation of MTO activity beyond the level achieved under adequate Cu supply was observed in a rat model of WD, suggesting Cu saturation of hepatic SELENBP1 under conditions of adequate Cu availability.

## 2 Materials and methods

### 2.1 Dietary TE deficiency in mice

Mice were held and fed as previously described [22]. Briefly, C57BL/6JRj mice were fed a chow diet for eleven weeks after birth. Thereafter, animals were subjected to TE-deficient diets, with five females and five males in each of the groups: control/adequate supply, Cu deficiency (-Cu), Se or Zn deficiency (-Se, -Zn, respectively), or combined deficiencies, -Cu/Se and -Cu/Se/Zn. Feeding occurred with food and water offered *ad libitum*. After eight weeks, the mice were sacrificed by CO_2_, blood was collected by cardiac puncture and their organs, including the liver, surgically dissected and immediately frozen. All procedures were performed in accordance with the institutional guidelines of Friedrich Schiller University Jena and the University Hospital Jena and approved by the Thuringian State Authority for Consumer Protection (permit number: FSU-20-003). TE concentrations in the samples were determined by total reflection X-ray fluorescence (TXRF) spectrometry, using a bench-top TXRF spectrometer (S4 T-STAR, Bruker Nano, Berlin, Germany) [23].

### 2.2 WD rat model

The ATP7B-deficient WD rat model was previously described [24,25]: Homozygous *Atp7b*^*−/−*^ rats progressively accumulate Cu in the liver, reaching a plateau at 70 days of age, whereas heterozygous *Atp7b*^*+/−*^ rats show normal intrahepatic Cu levels and thus serve as experimental controls [25]. *Atp7b*^*−/−*^ rats at 80-100 days of age were classified according to liver damage: animals affected by intrahepatic Cu accumulation showed serum aspartate aminotransferase (AST) and bilirubin levels below 200 U/L and 0.5 mg/dL, respectively, while animals with overt WD (diseased rats) had serum AST and bilirubin levels above 200 U/L and 0.5 mg/dL, respectively [24]. Liver homogenates from Atp7b^+/−^ (controls) and Atp7b^−/−^ (affected and diseased) rats of both sexes were used for the analyses. Rat experiments were done in accordance with the EU Animal Welfare Act and approved by the government authorities on animal care (permit numbers of the government of Oberbayern: AZ 55.2-2532.Vet_02_14_162 and AZ 55.2-2532.Vet_02_19_100).

### 2.3 Cell culture

HepG2 human hepatoma cells were obtained from the German Collection of Microorganisms and Cell Cultures (DSMZ, Braunschweig, Germany) and cultured as previously described [26] in Dulbecco's modified Eagle's medium (DMEM, low glucose; Sigma-Aldrich/Merck, Darmstadt, Germany, Cat# D6046), supplemented with 10% (v/v) fetal bovine serum (FBS; Biochrom, Berlin, Germany), 100 U/mL penicillin, 100 μg/mL streptomycin and non-essential amino acids (in MEM; Sigma-Aldrich, Cat# M7145). Cells were maintained at 37°C in a humidified atmosphere with 5% (v/v) CO_2_. No Cu was added beyond the Cu present in FBS. For Cu depletion experiments, cells were treated with the Cu chelator bathocuproinedisulfonate (BCS, Sigma-Aldrich, Cat# B1125) for 48 h. Cell lysates were prepared in homogenization buffer (100 mM Tris, 300 mM KCl, 0.1% Triton-X 100, pH 7.6) with subsequent sonication (50% output; 10 s pulse) and centrifugation (4°C; 16000×*g*; 10 min).

### 2.4 Cloning, bacterial overexpression and purification of murine SELENBP1

Recombinant murine SELENBP1 was obtained as previously described for human SELENBP1 [27]. In brief, the coding sequence of murine SELENBP1 was amplified by PCR from cDNA derived from visceral adipose tissue of C57BL/6J mice [28] and cloned first into the pASG-IBA102 vector (IBA Biosciences, Göttingen, Germany) and subsequently into the PURExpress Control DHFR plasmid (New England Biolabs, Ipswich, MA) to optimize protein expression. Murine SELENBP1 was produced in *E. coli* KRX bacteria (Promega, Madison, WI) as recombinant protein with a Twin-Strep tag (IBA Biosciences) attached at its C-terminus. Following bacterial lysis, the recombinant protein was purified using Strep-Tactin-XT 4Flow columns (IBA Biosciences), according to the manufacturer's instructions. Four more washing steps with 1 ml HBS containing 20 mM MgCl_2_ and 5 mM ATP were added. Where applicable, Cu(II) treatment was performed prior to protein elution from the affinity column by washing the column with 4 mL of HEPES-buffered salt solution (HBS; 50 mM HEPES, 150 mM NaCl, pH 7.4) containing 10 μM CuCl_2_, followed by five washes with 2 mL HBS. Recombinant proteins were eluted using HBS containing 50 mM biotin (IBA Lifesciences). The copper content of the purified protein was analyzed by TXRF as previously described [4].

### 2.5 Chemicals

If not stated otherwise, all chemicals were obtained from Carl Roth (Karlsruhe, Germany) or Sigma-Aldrich/Merck (Darmstadt, Germany).

### 2.6 Quantitative RT-PCR

Isolation of total RNA from murine liver samples, removal of genomic DNA, reverse transcription and real-time PCR were performed as previously described [23]. Murine *Selenbp1* (*NM_009150.3*) was amplified, using specific forward (5′-TGAGCCTCTGCTCGTTCC-3′) and reverse (5′-TGGACCACACTTTGTGCATT-3′) primers. Triplicates of all samples and standards were measured in the Mx3005P QPCR System (Agilent Technologies, Santa Clara, CA), with the following steps: 3 min at 95°C, 40 cycles of 15 s at 95°C, 20 s at 60°C, and 30 s at 72°C. Quantification was performed with standard curves from diluted PCR products. Sample values were normalized based on the reference genes *Hprt1* and *Rpl13a* [22].

### 2.7 Immunoblotting

Lysates were generated from liver tissues as previously described [23], and their protein concentrations were determined by Pierce™ BCA Protein Assay Kit (Thermo Fisher Scientific, Waltham, MA). SDS-PAGE and immunoblotting to nitrocellulose membranes (GE Healthcare, Chicago, IL) were performed according to standard procedures. After blocking, membranes were probed with antibodies against SELENBP1 (MBL Nagoya, Japan, #M061-3), or sulfide:quinone oxidoreductase (SQOR; Sigma-Aldrich, #HPA017079), followed by incubation with the appropriate horseradish peroxidase (HRP)-coupled secondary antibody (goat anti-mouse IgG, Thermo Fisher Scientific, #31430; or goat anti-rabbit IgG, Cell Signaling Technology, Danvers, MA, #7074) and detection with SuperSignal West Pico Substrate (Thermo Fisher Scientific) using a ChemiDoc™ MP analyzer (Bio-Rad Laboratories, Munich, Germany). Densitometric analyses were performed using Image Lab software (Bio-Rad). Relative protein levels were calculated after normalizing to Ponceau-S stained membranes [29].

### 2.8 MTO activity assay

MTO activity was determined using a coupled enzyme assay as previously described [4,27,29]. In brief, soluble protein fractions of liver or HepG2 lysates were deposited as duplicates on a 384-well plate, at final protein concentrations of 0.75 or 1 mg/mL, respectively. Recombinant proteins were used at 0.25 mg/mL. These MTO reaction mixes were applied adjacent to wells with methionine γ-lyase (MGL) reaction mixes. Recombinant MGL was produced as described [27]. MGL-mediated *in situ*-production of MT was initiated by adding methionine in HEPES-buffered saline (HBS; 50 mM HEPES, 150 mM NaCl, pH 7.4) to recombinant MGL in HBS, resulting in a final concentration of 20 mM methionine and 0.225 mg/mL MGL. Negative controls were set up on a separate 384-well plate as one technical replicate, where the MGL reaction mix contained only methionine without MGL. Plates were covered with an H_2_S indicator paper soaked in 20 mM Pb(II)-acetate after starting the MGL reactions. Pairwise comparison between the control group (‘ctrl’) and groups with different TE deficiencies was conducted on independent indicator papers. Cell, mouse and rat samples were incubated for 17, 16 and 4.5 h, respectively, at 37°C and under agitation at 150 rpm. Afterwards, black PbS spots were detected with a ChemiDoc MP analyzer and Image Lab software (Bio-Rad Laboratories; Munich, Germany) for assessment of MTO-generated H_2_S.

For detection of hydrogen peroxide (H_2_O_2_), 25 μL of the MTO assay reaction mixture (containing 6.25 μg recombinant protein or 18.75 μg liver lysate protein) was transferred to a black 384-well microplate after incubation of the MTO reaction for H_2_S detection. H_2_O_2_ was detected using the Amplex™ Red Hydrogen Peroxide/Peroxidase Assay Kit (Thermo Fisher Scientific) according to the manufacturer's instructions. Fluorescence was recorded using a CLARIOstar microplate reader (BMG LABTECH, Ortenberg, Germany) at 571 nm (ex)/585 nm (em).

For detection of formaldehyde, 4-amino-3-hydrazino-5-mercapto-1,2,4-triazole (Purpald reagent) was used. Under alkaline conditions, Purpald reacts with aldehydes to form a reaction product that can be detected photometrically [30]. Following incubation of the MTO reaction for detection of H_2_S, 30 μL of the reaction mixture (containing 7.5 μg of recombinant protein or 22.5 μg of liver lysate protein) was transferred to a transparent 384-well microplate. Subsequently, 15 μL of 68.4 mM Purpald reagent, prepared in 1 M NaOH, was added. The reaction mixture was incubated for 15 min at room temperature, followed by photometric detection at 549 nm using a SPECTROstar Nano microplate reader (BMG LABTECH).

### 2.9 Statistical analysis

Data are expressed as means + SD, unless stated otherwise. All calculations were performed using GraphPad PRISM, version 9 and up (GraphPad Software, San Diego, CA). Statistical analysis was done either using a two-tailed unpaired *t*-test with Welch's correction or using a Kruskal-Wallis test with Dunn's multiple comparisons test as denoted in the respective Figure legends.

## 3 Results and discussion

### 3.1 Murine SELENBP1 is a copper-binding MTO

In order to assess MTO activity of murine SELENBP1, we tested the recombinant protein for MTO activity and compared it to human SELENBP1; we also tested whether Cu, as expected for the human protein [4], stimulates that activity. As demonstrated in Fig. 1C, recombinant murine SELENBP1, like its human homolog, is an MTO, as determined by the generation of H_2_S in a coupled MTO assay based on *in situ*-generation of methanethiol (Fig. 1D). Moreover, the addition of Cu(II) to the protein further enhanced its enzymatic activity (Fig. 1C, blue bars). In line with these findings, the addition of Cu(II) during protein preparation, followed by washing and Cu content analysis by TXRF, resulted in Cu bound to both proteins, at a molar ratio of approx. 1 Cu per SELENBP1 molecule (Fig. 1E).

### 3.2 Cu-deficient diets provoke a decrease in hepatic MTO activity in mice

In mammals, the liver represents the hub for homeostasis and metabolism of essential TEs, harboring high levels of Cu and Se for production and (in part) secretion of large amounts of Cu- and Se-containing proteins at adequate dietary supply [1,19]. SELENBP1, which is highly expressed in the liver [6], is unique in binding both Cu and Se [4], although it was originally named according to its Se-binding capability [3]. Orthologs of human SELENBP1 in the rat and in the plant model organism *Arabidopsis thaliana* have also been reported to bind Zn [16,31], another essential TE that may serve as cofactor in enzymes [20]. In the present study, we analyzed liver samples obtained from mice that had received diets deficient in Cu, Se and/or Zn for eight weeks [22], exploring whether dietary availability of these TEs affects the enzymatic (MTO) activity of hepatic SELENBP1. Fig. S1 shows replotted data from Ref. [22], summarizing the outcome of the TE-deficient diets on Cu, Se and Zn contents in the murine liver, with emphasis on differences between female and male mice.

Intrahepatic levels of Cu were similar in female and male mice adequately supplied with TEs, while females showed slightly higher Se levels, as compared to males, under these conditions. All dietary interventions that included Cu and/or Se deficiency (-Cu, -Se, -Cu/Se and -Cu/Se/Zn) resulted in statistically significant decreases in the intrahepatic levels of the respective TEs by approximately 50% (Fig. S1), in accordance with previous studies in mice, reporting Cu and Se depletion of the liver in response to dietary deficiency [1,32]. We also noticed some interplay between the three explored TEs in a sex-specific manner, such as a slight but statistically significant decrease in hepatic Cu and Se levels upon Zn deficiency (-Zn) solely in female mice (Fig. S1).

**Fig. 2:**
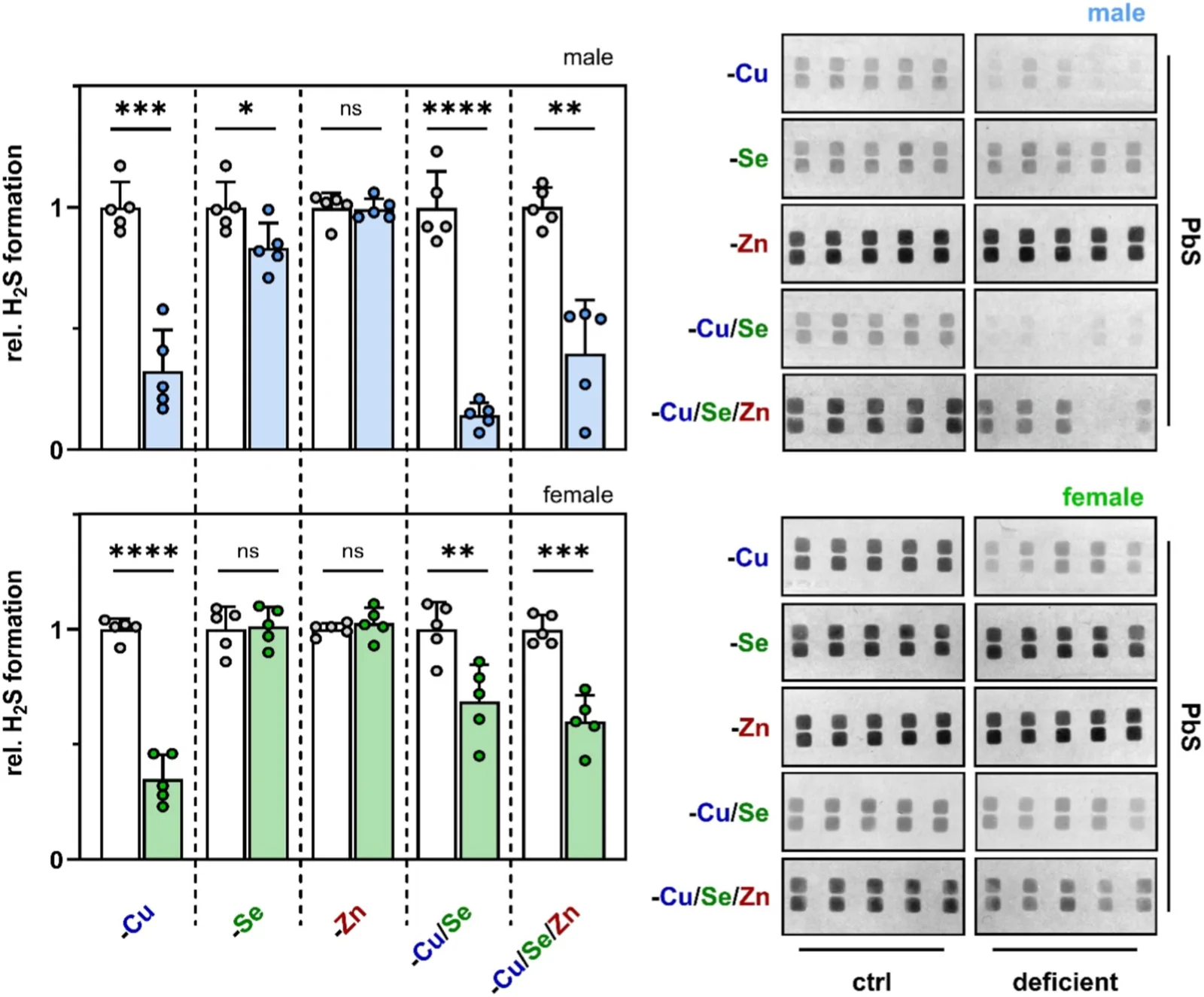
Cu-deficient diets decrease hepatic MTO activity in mice. MTO activity in liver lysates was determined by measuring methanethiol-derived H_2_S production in a coupled enzyme assay. Comparison of all intervention groups (left: blue/green bars; right: “deficient”) with the control group (left: white bars; right: “ctrl”) was performed on separate indicator papers. Pixel density of the obtained black PbS spots were quantified by densitometric analysis (n = 5 for males (upper panel) and females (lower panel)). Statistical significance was tested using a two-tailed unpaired *t*-test with Welch's correction (ns, not significant, *p < 0.05, **p < 0.01, ***p < 0.001, and ****p < 0.0001 *vs.* adequate).

Based on our recent findings on the Cu requirement of recombinant human SELENBP1 for its MTO activity [4], we here sought to investigate the effect of dietary Cu availability on hepatic MTO activity. To this end, we assessed hepatic MTO activity *ex vivo*, employing a previously established coupled MTO assay [27], determining the production of H_2_S from liver lysates exposed to MT generated *in situ* using the MT-generating enzyme, MGL (Fig. 1D). All dietary interventions that included Cu deficiency (-Cu, -Cu/Se, -Cu/Se/Zn) resulted in statistically significant decreases in hepatic MTO activity in both male and female mice, as compared to the adequately supplied controls (Fig. 2, Fig. S2). In mice of both sexes, dietary Cu deficiency alone (-Cu groups) was sufficient to lower hepatic MTO activity to ∼35%. In mice with combined Cu and Se deficiency (-Cu/Se groups), hepatic MT-derived H_2_S generation was diminished to 69% in females and to 14% in males, respectively. In the -Cu/Se/Zn groups, MTO activity in liver samples decreased to 40% in males and to 60% in females, respectively. Whereas a modest yet statistically significant decline in hepatic MTO activity was also detected in male mice fed a Se-deficient diet, no alterations in MTO activity were detected in livers from Se-deficient females. Zn deficiency (-Zn) alone did not alter hepatic MTO activity (Fig. 2).

**Fig. 3:**
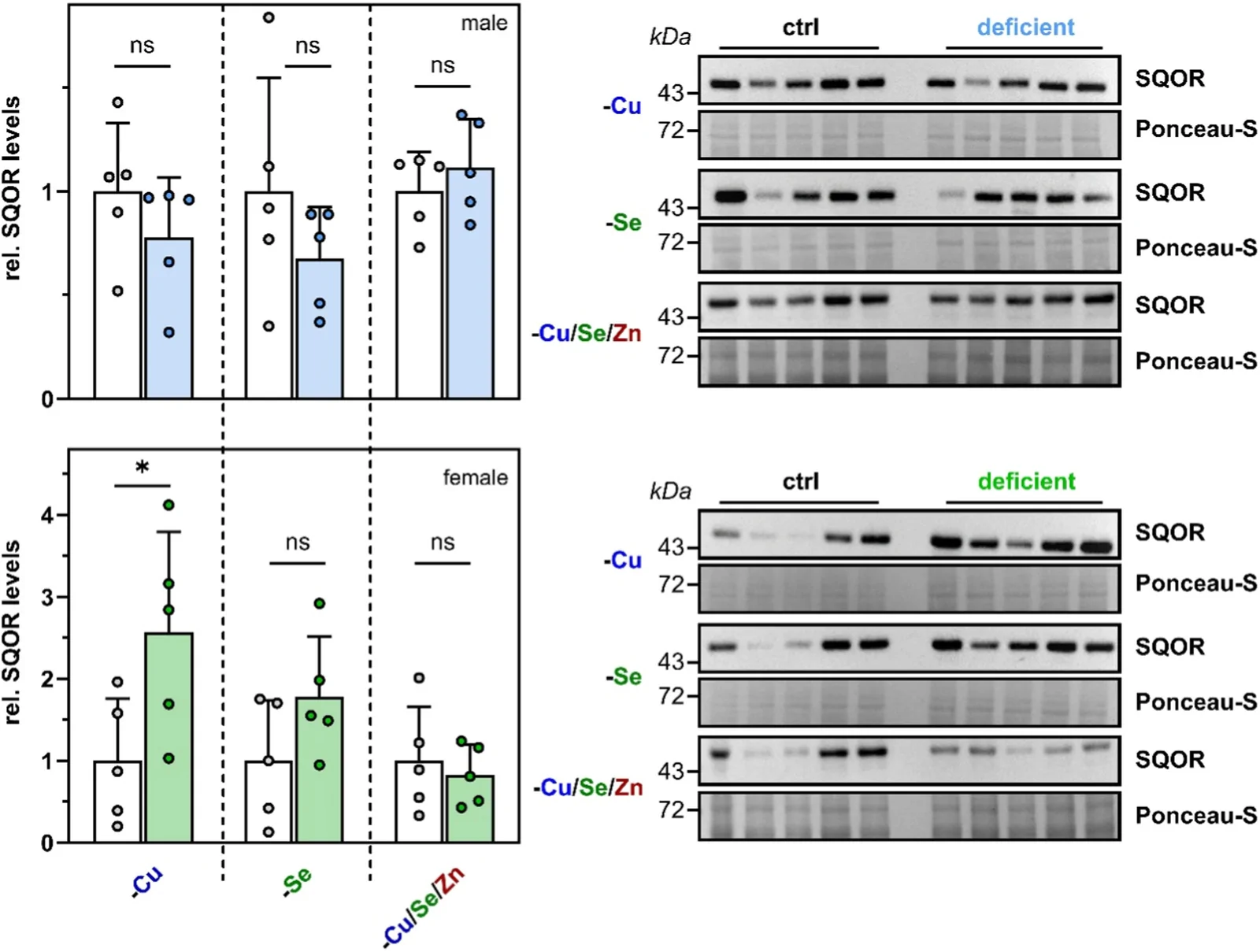
Protein levels of the rate-limiting enzyme for H_2_S catabolism, SQOR, in the liver of mice subjected to selected TE-deficient diets. SQOR was detected in liver lysates of male (upper panel) and female (lower panel) mice by immunoblotting. Band intensity was quantified by densitometric analysis and normalized to Ponceau-S staining. Data are depicted as means + SD relative to the adequately supplied control groups (n = 5). Statistical significance was tested using a two-tailed unpaired *t*-test with Welch's correction (ns, not significant, *p < 0.05 *vs.* ctrl).

As H_2_S is efficiently catabolized through the mitochondrial sulfide oxidation pathway in many mammalian tissues, including the liver [33], a parallel modulation of the first and rate-limiting enzyme for H_2_S oxidation, SQOR [29,33], by the respective dietary intervention could potentially interfere with the MTO assay. Detection of other MTO products, H_2_O_2_ and formaldehyde, works well with recombinant protein, but, likely owing to their rapid degradation, is not suitable for the analysis of liver lysates (Fig. S3). We therefore tested whether SQOR protein levels were altered in selected samples (-Cu, -Se, and -Cu/Se/Zn groups, as compared to adequately supplied mice); however, neither single dietary deficiency of Cu and Se nor combined deficiency of Cu, Se and Zn resulted in statistically significant alterations, with the exception of a slight increase in SQOR levels that was observed in the liver of female mice fed Cu- and Se-deficient diets (Fig. 3). Thus, the observed changes in H_2_S levels (Fig. 2) result from altered MTO activities.

Taken together, these data indicate that adequate dietary Cu supply is essential for the MTO activity of hepatic SELENBP1 and thus the capability of the liver to oxidize MT. SELENBP1 was recently added to the list of human cuproenzymes [18,19], based on our previous findings regarding the requirement of Cu for the MTO activity of recombinant human SELENBP1 *in vitro* [4]. The data presented here demonstrate the relevance of Cu for the enzymatic function of mammalian SELENBP1 in a mouse model of dietary TE deficiency, in line with previous reports on SELENBP1 orthologs in nematodes and in bacteria [4,34]. They also support the notion that Se is of minor importance and Zn is not required as cofactor, as previously established for recombinant human SELENBP1 *in vitro* [4].

**Fig. 4:**
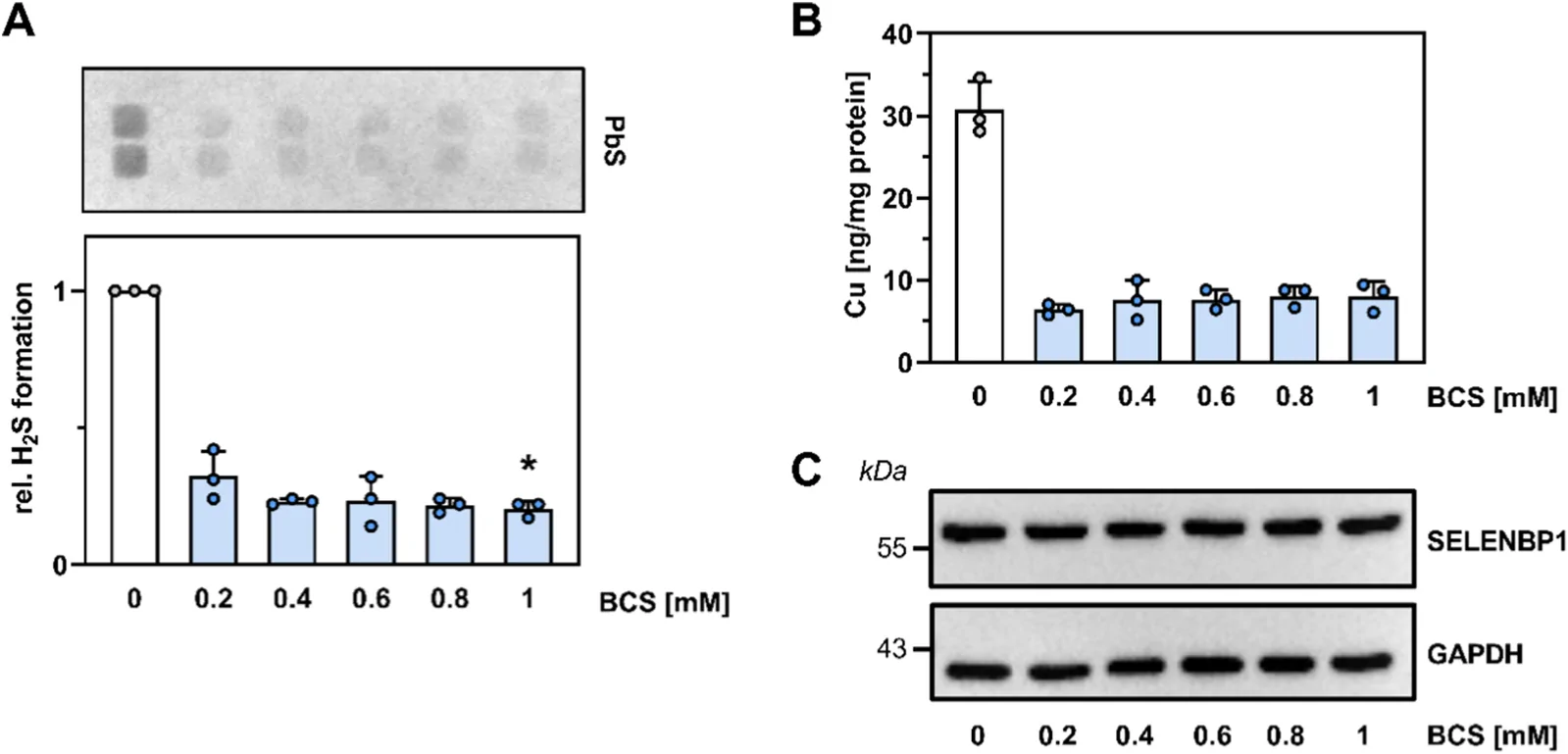
Effect of Cu depletion on MTO activity, Cu levels and SELENBP1 protein levels in HepG2 hepatoma cells. HepG2 cells were treated with BCS at the indicated concentrations for 48 h, followed by lysis in homogenization buffer (100 mM Tris, 300 mM KCl, 0.1% Triton-X 100). Cell lysates were checked for **(A)** MTO activity by measuring methanethiol-derived H_2_S production in a coupled enzyme assay, **(B)** Cu content by TXRF and **(C)** SELENBP1 protein levels by immunoblotting. Statistical significance was tested using Kruskal-Wallis test with Dunn's multiple comparisons test (*p < 0.05 *vs.* 0 mM BCS).

Furthermore, the importance of Cu as cofactor for MTO was underpinned by culturing HepG2 human hepatoma cells in the presence of the Cu chelator BCS: exposure to BCS for 48 h lowered intracellular Cu levels, accompanied by suppression of MTO activity by ∼70-80%, while SELENBP1 levels remained unaltered (Fig. 4).

Similar to selenoproteins, a hierarchy might be postulated for cuproproteins: the hierarchy concept for selenoproteins refers to the observation that the levels of some selenoproteins remain largely stable under selenium scarcity, whereas others are less synthesized and/or being degraded under these conditions [1]. Similarly, it could be assumed that, under conditions of limited dietary supply, Cu will be preferentially shifted to those cuproenzymes that are most relevant for the proper functioning of the cell and the organism, such as mitochondrial cytochrome *c* oxidase [18,19]. As the mechanisms of selenoprotein and cuproprotein biosynthesis differ drastically, with cotranslational incorporation of selenocysteine in the former and predominantly posttranslational incorporation of Cu in the latter, the hierarchy concept for selenoproteins cannot be simply transferred to cuproproteins, and therefore an analogous concept for cuproproteins remains speculative. Nevertheless, it is conceivable that abundant Cu-dependent enzymes with key metabolic functions compete with SELENBP1 for the available Cu. The observation that dietary Cu deficiency translates into decreased MTO activity (Fig. 2) suggests that SELENBP1 would not be ranked high in an assumed cuproprotein hierarchy.

Regarding liver pathologies, Cu deficiency has been found to be relatively frequent in patients with advanced cirrhosis [35]. This may result in impaired hepatic MTO activity, and indeed, elevated MT concentrations have been measured in the blood of patients who suffered from cirrhosis as a consequence of alcoholic liver disease [14]. Moreover, the breath of cirrhotics has been shown to contain elevated levels of dimethyl sulfide (DMS), a volatile MT derivative that is a characteristic component of *fetor hepaticus*, a malodor of patients with advanced liver diseases [36]. DMS is generated through methylation of MT, if SELENBP1 is downregulated or its MTO activity is diminished [6,7].

### 3.3 Hepatic SELENBP1 levels are altered in a sex-specific manner in mice under dietary TE deficiency

In addition to MTO activity, we analyzed mRNA and protein levels of SELENBP1 in liver samples obtained from mice held on TE-deficient diets, and their control counterparts with adequate dietary TE supply.

**Fig. 5:**
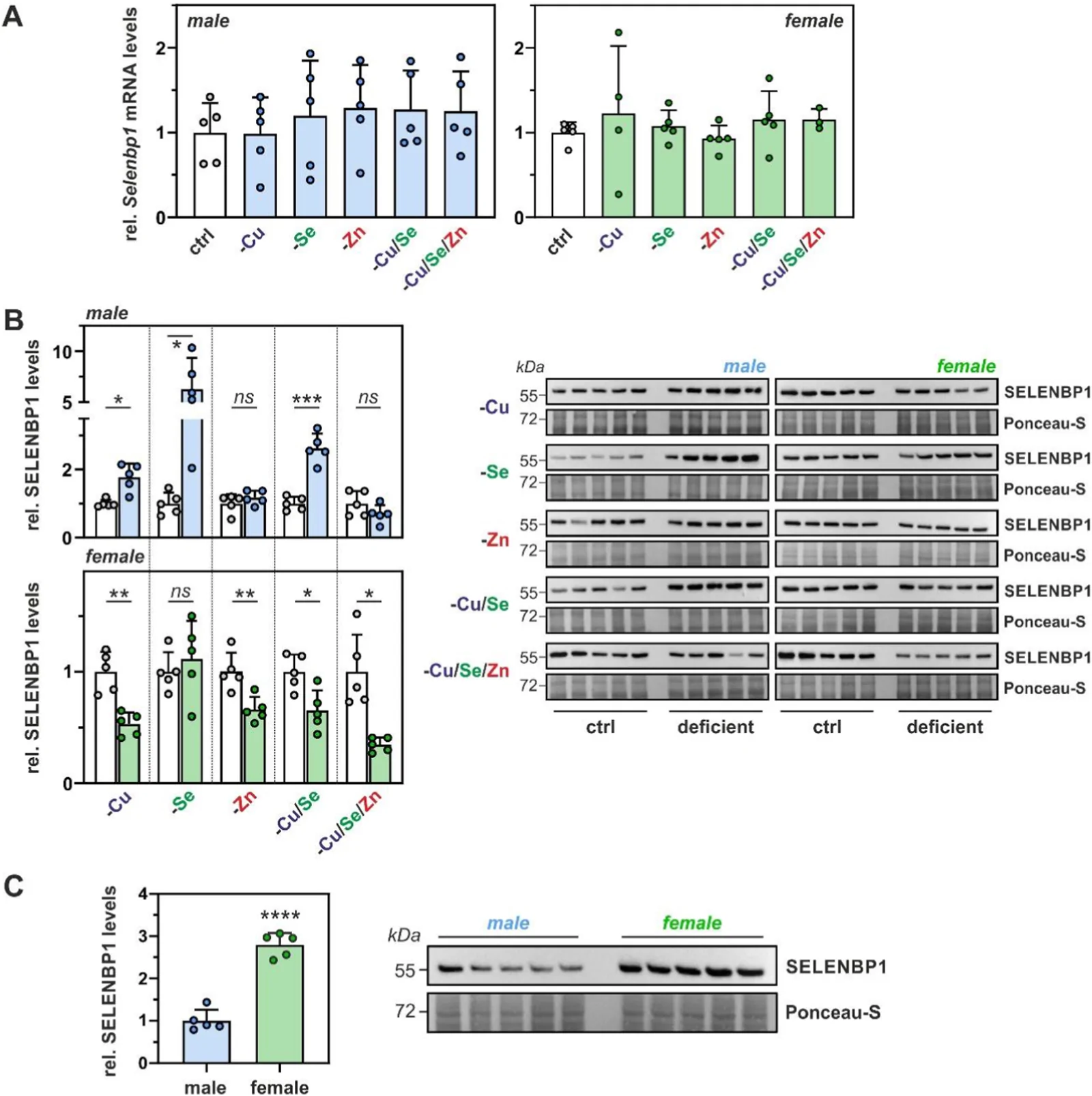
SELENBP1 mRNA and protein levels in the liver of mice subjected to diets deficient in Cu, Se and/or Zn. (A) Se*lenbp1* mRNA levels were determined by qPCR. PCR products were quantified with a standard curve and normalized to the housekeeping genes *Hprt1* and *Rpl13a*. Data are given as relative levels (means + SD), with male (left panel) and female (right) mice analyzed separately (n = 3-5/group). **(B, C)** SELENBP1 protein levels were determined by immunoblotting of the liver lysates. Comparison of all intervention groups with the respective control group (ctrl) was performed on separate membranes. Band intensity was quantified by densitometric analysis and normalized to Ponceau-S staining. Data in B are depicted as means + SD relative to the control groups with adequate TE supply (n = 5 for males and females); in C, samples from male and female ctrl animals with identical overall protein content were analyzed on the same blot and densitometry data depicted as means + SD. B and C: Statistical significance was tested using a two-tailed unpaired *t*-test with Welch's correction (ns, not significant, *p < 0.05, **p < 0.01, ***p < 0.001, and ****p < 0.0001 *vs.* adequate (white bars, B) or *vs*. male (C)).

Of note, mice, unlike humans and rats, possess two closely related SELENBP/MTO proteins, which are almost 98% identical (Fig. 1) [37] and therefore hardly distinguishable; we thus expect our SELENBP1-specific primers and antibody to equally well detect any SELENBP2 in murine tissue lysates. At the transcript level, no alteration of *Selenbp1* gene expression was observed in the liver of female or male mice subjected to any of the TE-deficient dietary conditions (Fig. 5A). In contrast, hepatic SELENBP1 protein levels were altered in a sex-specific manner: in male mice, statistically significant increases were observed in response to Cu-, Se- and combined Cu/Se-deficient diets, whereas no significant alterations were detected under diets with Zn deficiency included (-Zn and -Cu/Se/Zn groups). The highest increase occurred in response to the diet solely deficient in Se, with 6-fold elevated SELENBP1 levels, as compared to the adequately supplied control group. In female mice, all dietary interventions except Se deficiency resulted in statistically significant decreases in hepatic SELENBP1 protein levels, to as low as 53% (-Cu), 66% (-Zn), 65% (-Cu/Se) or 35% (-Cu/Se/Zn) of control conditions (Fig. 5B).

Very few reports on sex-specific differences in the regulation of gene expression and protein levels in response to dietary TE deficiencies exist; for example, both pre- and posttranscriptional mechanisms were discussed as underlying the differences in regulation of the selenoenzyme, iodothyronine deiodinase 1 (DIO1), in liver and kidneys of Se-replete and Se-deficient female and male mice [38]. As no changes in mRNA levels were detected, post- or non-transcriptional mechanisms appear to underlie the observed differences in SELENBP1 levels between male and female mice in response to TE deficiencies.

Comparing liver samples of male and female control mice, we also noticed that SELENBP1 protein levels were nearly threefold higher in female than in male mice (Fig. 5C). This is, however, not unusual, as the liver has been reported to show the highest degree of sex-dependent variability among all mammalian tissues, with 72% of the genes differentially expressed between females and males [39]. Sex-specific differences in hepatic gene and protein expression are mainly related to steroid, energy and xenobiotic metabolism, and their impact on development and progression of liver diseases is being increasingly recognized [39,40]. Higher hepatic mRNA and/or protein levels were reported for female rodents, as compared to males, for several proteins involved in TE homeostasis, such as the Cu- and Zn-binding thioneins, the hepatic Cu exporter, ATP7B, the liver-secreted plasma Se transport protein selenoprotein P (SELENOP) and the selenoenzyme DIO1 [38,[41], [42], [43]].

Altogether, our data suggest that the outcome of dietary TE, and in particular Cu, deficiency on hepatic MTO activity in mice is shaped by a sexual dimorphism. Even though Cu deficiency resulted in diminished MTO activity in both sexes, the molecular mechanisms by which low intrahepatic Cu levels decrease the MTO activity appear to be somewhat different between females and males: In male mice, the increased SELENBP1 protein levels under Cu-deficient conditions may manifest an attempt of the liver to compensate for the lowered hepatic MTO activity that is due to insufficient Cu availability. In female mice, low hepatic MTO activity under Cu-deficient conditions may result from both impaired Cu binding and decreased SELENBP1 protein levels.

### 3.4 Excessive intrahepatic Cu accumulation does not affect MTO activity as long as the liver is not severely damaged

In order to assess whether not only a Cu deficit but also Cu excess may affect hepatic MTO activity, we analyzed liver samples obtained from rats carrying a deletion in the *Atp7b* gene. The resulting non-functional hepatic Cu exporter ATP7B causes a WD-like phenotype in homozygous *Atp7b*^*−/−*^ rats, whereas heterozygous *Atp7b*^*+/−*^ rats show no abnormalities [25]. Even though all of the *Atp7b*^*−/−*^ rats develop progressive hepatic Cu accumulation, two subgroups are distinguishable at 80-100 days of age: “affected” animals with minor impairment of their liver function and “diseased” animals with severe liver damage [24].

**Fig. 6:**
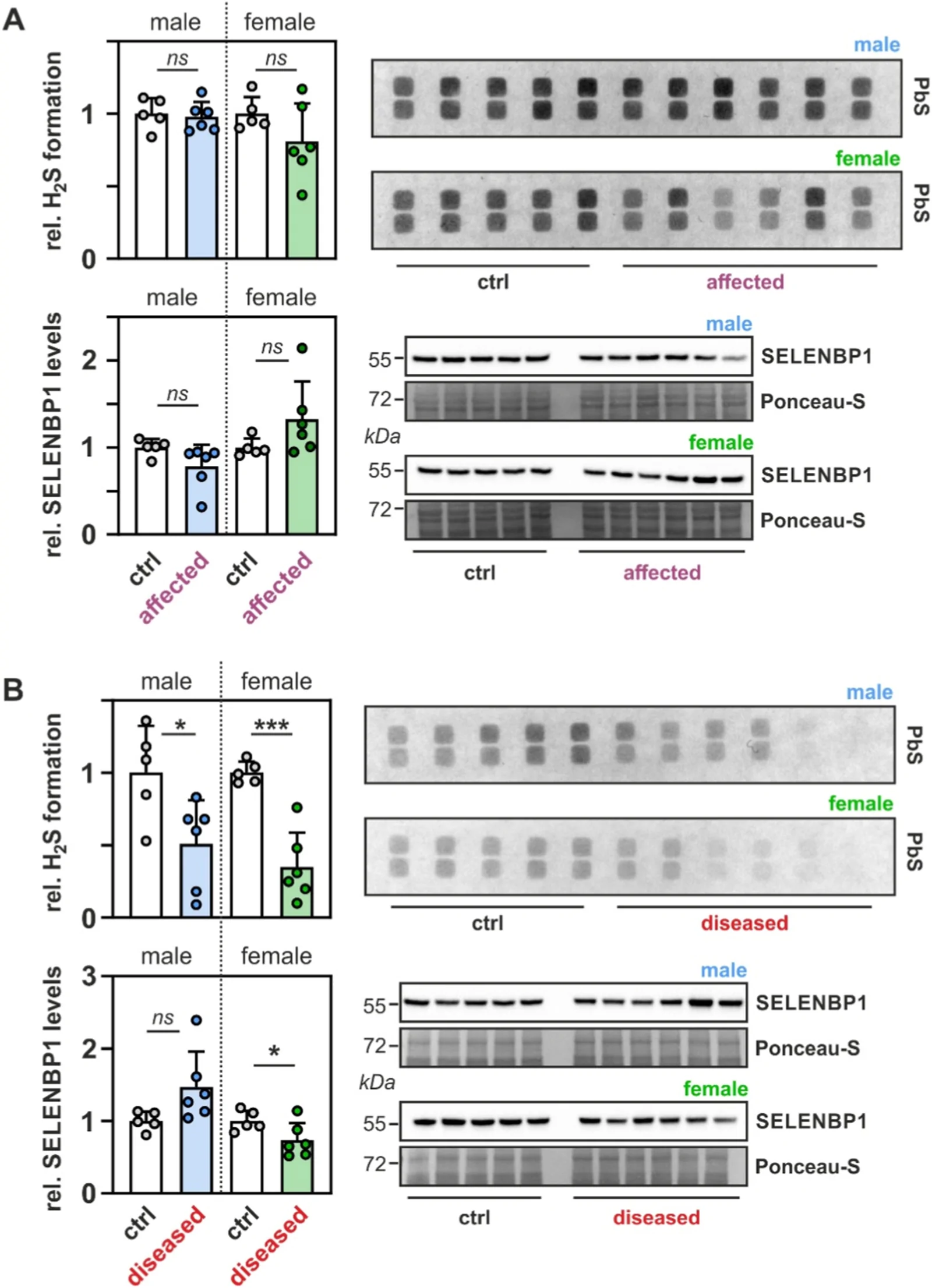
Excessive hepatic Cu accumulation does not further increase MTO activity. Liver lysates from rats carrying a WD-like inactivating mutation in the *ATP7b* gene were analyzed for SELENBP1 levels and MTO activity. Samples from (unaffected) heterozygous (*Atp7b*^+/−^) rats were taken as control (ctrl). Samples from homozygous rats (*Atp7b*^−/−^) were distinguished according to the extent of liver damage: affected **(A)** or diseased **(B)** male (blue) and female (green) rats were analyzed. MTO activity and SELENBP1 levels were determined as described in Fig. 2, Fig. 5, respectively. Data are depicted as mean + SD relative to the control groups (control n = 5; affected n = 6; diseased n = 6). Statistical significance was tested using a two-tailed unpaired *t*-test with Welch's correction (ns, not significant, *p < 0.05, ***p < 0.001 *vs*. ctrl).

Affected female and male rats showed no statistically significant alterations in hepatic MTO activity, as compared to the heterozygous *Atp7b*^*+/−*^ control rats. Similarly, no differences in hepatic SELENBP1 protein levels were observed in affected *vs*. control rats (Fig. 6A).

On the other hand, diseased rats of both sexes exhibited pronounced and statistically significant decreases in hepatic MTO activity, with activities lower by ∼50% and ∼65% in males and females, respectively, relative to controls (Fig. 6B). Differences in SELENBP1 protein levels were reminiscent of the sexual dimorphism in mice (Fig. 5), with a statistically significant decrease in females but not in males, as compared to the respective controls (Fig. 6B). The decrease in MTO activity in the liver samples of the diseased rats is most likely due to loss of functional hepatocytes, as excess intrahepatic Cu accumulation is known to impair metabolic processes and to induce mitochondrial dysfunction, oxidative and ER stress in the liver [21,[44], [45], [46]]. Moreover, this assumption is consistent with observations made in patients with advanced liver disease who showed elevated blood levels of MT [[12], [13], [14]] that might be attributable to an impaired hepatic MT oxidation.

Taken together, these data suggest that increasing the intrahepatic Cu levels above physiological levels does not further enhance MTO activity, indicating Cu saturation of SELENBP1 under conditions of adequate dietary TE supply.

## 4 Conclusions

Enzymatic (MTO) activity of recombinant human SELENBP1 has previously been shown to be Cu-dependent *in vitro* [4]. Here, we demonstrate that adequate dietary Cu supply is necessary and sufficient to support the MTO-catalyzed conversion of MT to H_2_S in the liver of mice. These data are in line with previous findings from non-mammalian organisms, such as *C. elegans*, where MTO activity of the SELENBP1 ortholog SEMO-1 [15] depends on Cu availability [4]. On the other hand, excessive intrahepatic Cu accumulation did not further augment hepatic MTO activity in WD-affected *Atp7b*^*−/−*^ rats, suggesting Cu saturation of SELENBP1 under physiological conditions and adequate dietary Cu supply.

The suppression of hepatic MTO activity in mice with dietary Cu deficiency and in WD-diseased *Atp7b*^*−/−*^ rats may stimulate and reinforce investigations on the suitability of MT as a non-invasive biomarker of severe disturbances in Cu homeostasis as well as severe liver damage, *e.g.*, in WD, cirrhosis and hepatocellular carcinoma. Although steady-state levels of MT in humans will vary not only with hepatic MTO activity, but also other factors, such as any changes in microbiome composition that may affect MT generation, the general feasibility of such an approach, based on detecting MT modifications of serum proteins, was demonstrated previously. Grigoryan et al. found an association of Cys34 S-methanethiolation of serum albumin with colorectal cancer in serum samples of colorectal cancer patients, relative to controls [47].

Finally, some methodological constraints of our study should be noted: (i) The detection of methanethiol-derived H_2_S by the coupled enzyme assay we applied here is laborious. Therefore, a limited number of 5-6 animals in each group were included for the analyses. Only recently, a more straightforward approach for an MTO assay was developed [48] so that, in future studies, this restriction is likely to be obsolete. (ii) The assessment of hepatic MTO activity relies solely on detection of volatile H_2_S, as the two other products of methanethiol oxidation, H_2_O_2_ and formaldehyde, were hardly detectable in the liver lysates in our setup. This is likely due to their rapid conversion through hepatic enzymes. (iii) We cannot exclude that some of the generated H_2_S escaped the detection by our assay, owing to metabolization by the enzyme SQOR. (iv) A potential threshold limit value of dietary copper supply for supporting optimal MTO activity in the liver (as well as in other tissues such as the large intestine) remains to be determined in future studies.

## CRediT authorship contribution statement

**Niklas Krafczyk:** Writing – review & editing, Writing – original draft, Visualization, Validation, Methodology, Investigation, Formal analysis, Conceptualization. **Kristina Lossow:** Writing – review & editing, Validation, Methodology, Investigation, Formal analysis, Conceptualization. **Lara Biegel:** Writing – review & editing, Investigation. **Nora Meinhardt:** Writing – review & editing, Investigation. **Maria Schwarz:** Writing – review & editing, Investigation. **Hans Zischka:** Writing – review & editing, Resources. **Holger Steinbrenner:** Writing – review & editing, Writing – original draft, Supervision, Conceptualization. **Anna Patricia Kipp:** Writing – review & editing, Supervision, Resources, Project administration, Funding acquisition, Conceptualization. **Lars-Oliver Klotz:** Writing – review & editing, Writing – original draft, Visualization, Supervision, Project administration, Funding acquisition, Conceptualization.

## Ethics declaration

This study was conducted in accordance with the ARRIVE (Animal Research: Reporting of In Vivo Experiments) guidelines. This study was approved by the Thuringian State Authority for Consumer Protection. (Approval No. FSU-20-003)

## Declaration of competing interest

The authors declare the following financial interests/personal relationships which may be considered as potential competing interests:Lars-Oliver Klotz reports financial support was provided by German Research Foundation. Given his role as a member of the Editorial Board of “Redox Biology”, Lars-Oliver Klotz had no involvement in the peer review of this article and had no access to information regarding its peer review. Full responsibility for the editorial process for this article was delegated to another journal editor. If there are other authors, they declare that they have no known competing financial interests or personal relationships that could have appeared to influence the work reported in this paper.

## Data Availability

Data will be made available on request.
