# Supplementary material for: Hepatic methanethiol oxidase (MTO) activity of mice requires adequate dietary supply of copper
Source: Redox Biol. 2026 Sep 6;97:104384. doi: 10.1016/j.redox.2026.104384 (PMC13579460; doi:10.1016/j.redox.2026.104384)
Supplement: Supplementary file 1 [file mmc1.pdf]

Supplementary Figures

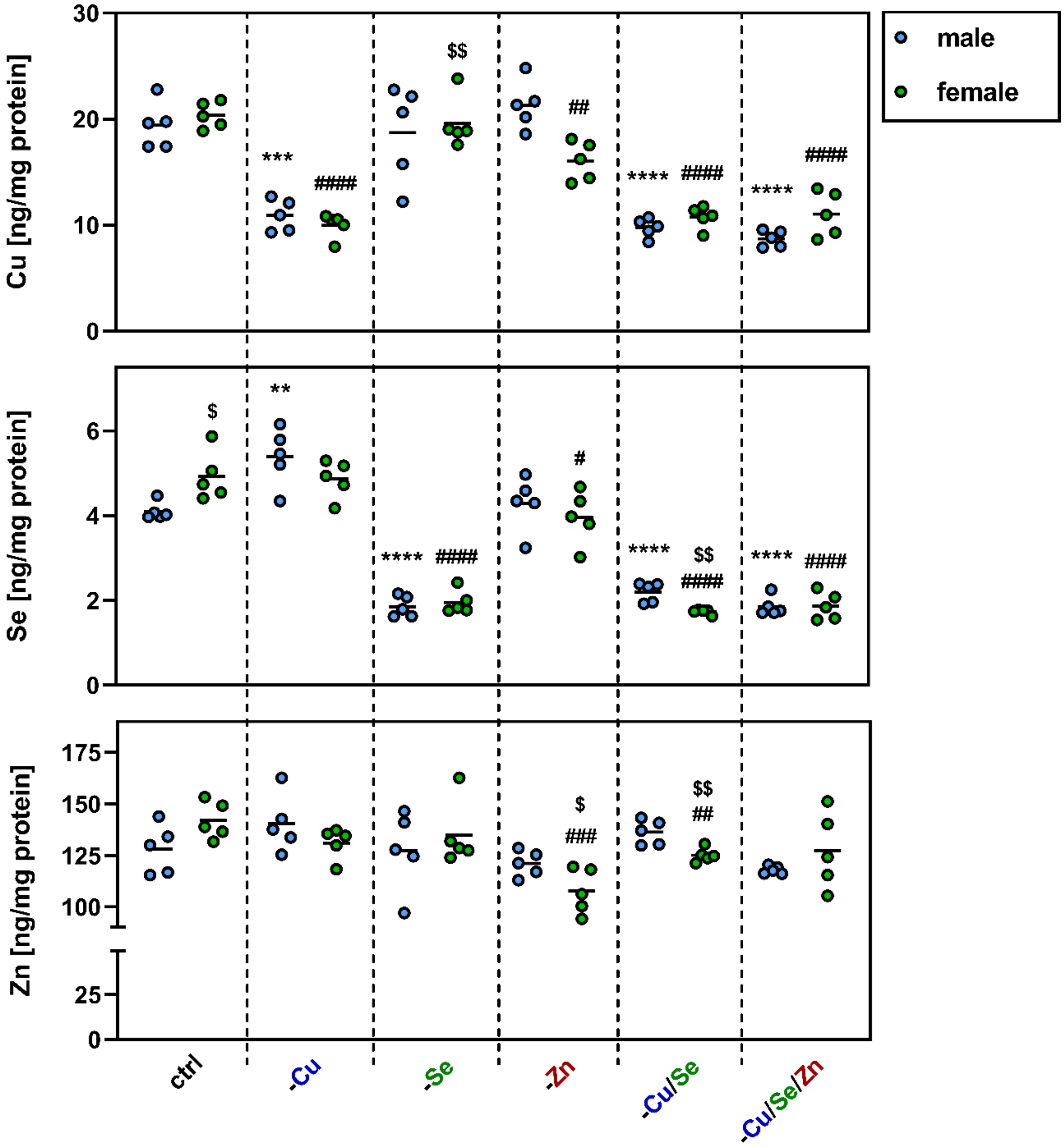

Figure S1. Dietary intervention to induce deficiencies of Cu, Se and/or Zn in mice. Trace element concentrations in the liver of C57BL/6Jrj mice after eight weeks of dietary Cu, Se and/or Zn deficiency. Samples of dissected liver tissues were lysed in RIPA buffer (50 mM Tris, 150 mM NaCl, 2 mM EDTA, 0.5% sodium deoxycholate, 0.1% SDS, 1% NP-40 alternative) and centrifuged (10 min, 14000 rcf, 4°C). Thereafter, the supernatants were collected, and the contents of trace elements were determined by total reflection X-ray fluorescence spectrometry (TXRF). Female and male mice were plotted and analyzed separately for each dietary group. Scatter dot plots with means (n=5). Statistical significance was determined by unpaired *t*-test ($^{**}p<0.01$, $^{***}p<0.001$ and $^{****}p<0.0001$ *vs.* adequately supplied males; $^{\#\#}p<0.01$, $^{\#\#\#}p<0.001$ and $^{\#\#\#\#}p<0.0001$ *vs.* adequately supplied females; $^{\$}p<0.05$ and $^{\$\$}p<0.01$ *vs.* the respective male intervention group).

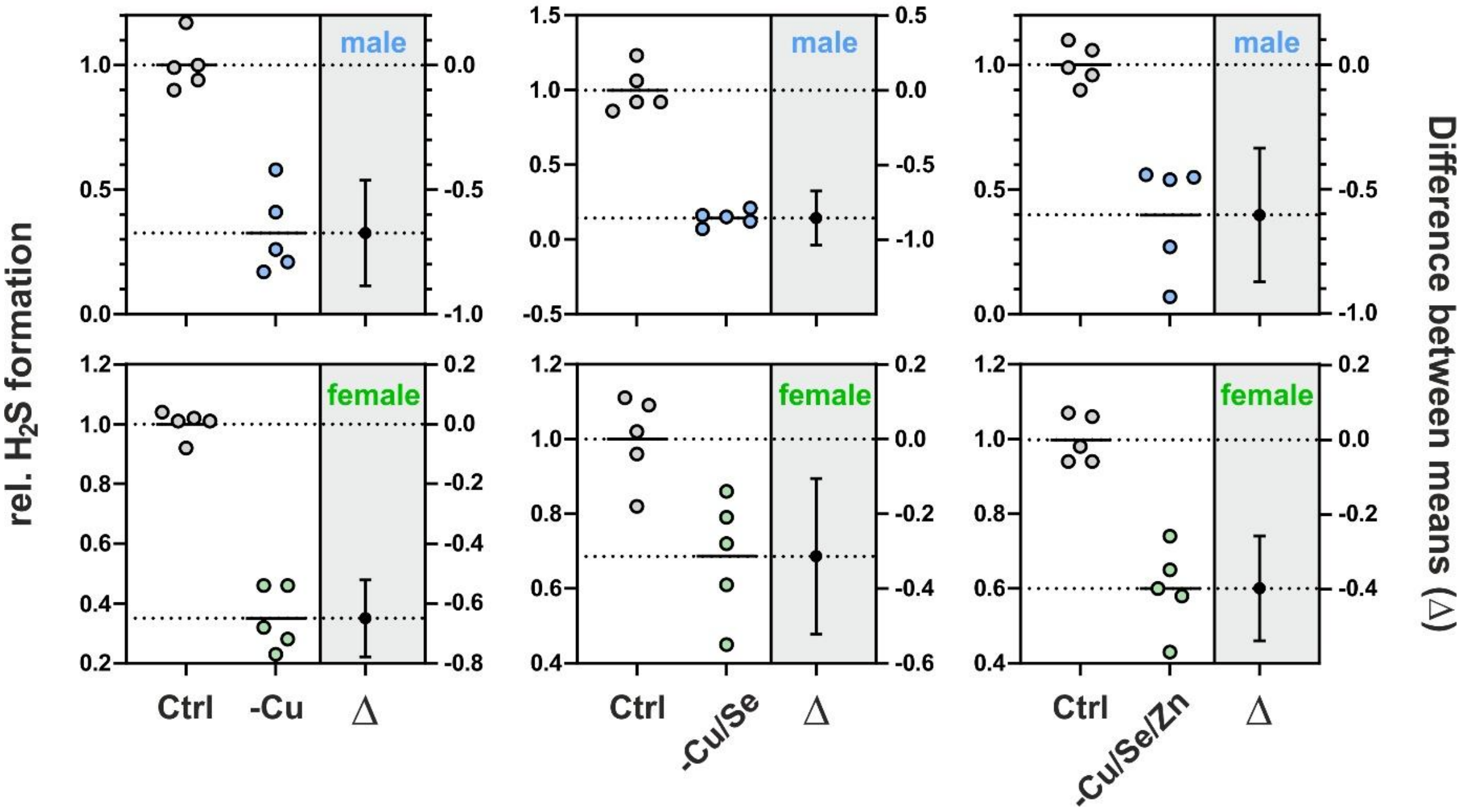

Figure S2. Cu-deficient diets decrease hepatic MTO activity in mice: effect size. Data from Figure 2, focusing on Cu-deficient diets, were used to generate estimation plots. Effect sizes were calculated as differences (Δ) between the two means (right y-axis). Error bars represent 95% confidence intervals.

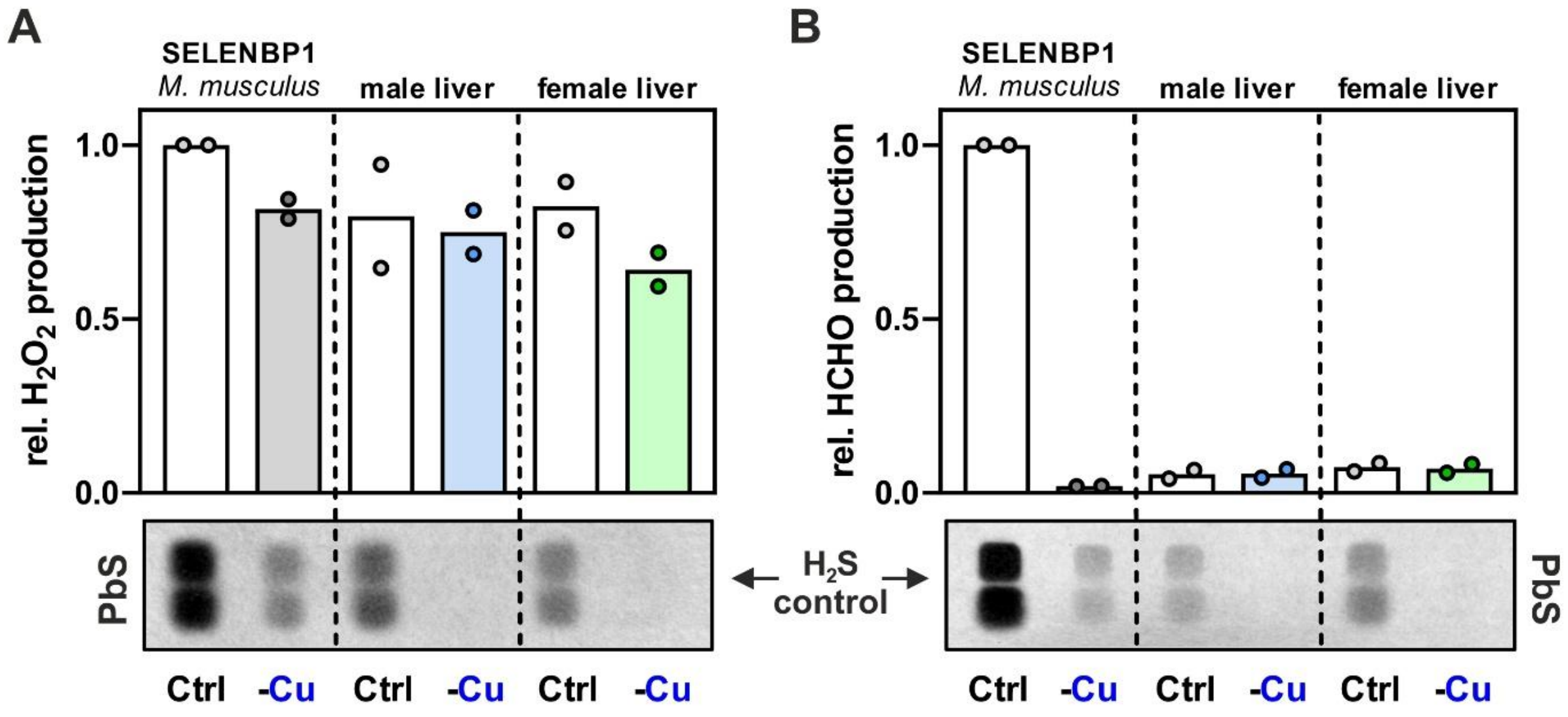

Figure S3. Detection of methanethiol oxidase (MTO) products in samples with recombinant protein and lysates. The figure shows the effect of Cu on methanethiol oxidase (MTO) activity parameters as determined with recombinant Strep-tagged murine SELENBP1 (left; bars 1-2) and in murine liver lysates (middle and right; bars 3-6). MTO activity was determined by measuring the release of **(A)** H_2O_2 (bar graph) or **(B)** formaldehyde (HCHO); H_2S release was tested from the same samples and lead sulfide (PbS) signals of one representative experiment are shown underneath the bar graphs. Two independent experiments were performed in duplicate each.

Whereas the effect of Cu (or Cu-deficiency) on MTO activity as seen in MTO assays using H_2S detection is replicated with H_2O_2 or formaldehyde when using recombinant enzyme, no clear differences between control (Ctrl) and Cu-deficient (-Cu) conditions are detectable in lysates. Hydrogen peroxide and formaldehyde signals are close to background signals.
